# Probiotics Enhance Cereal Yield and Quality and Modify Agrochemical Soil Properties

**DOI:** 10.3390/microorganisms10071277

**Published:** 2022-06-23

**Authors:** Virgilija Gavelienė, Sigita Jurkonienė

**Affiliations:** Nature Research Centre, Institute of Botany, Laboratory of Plant Physiology, Akademijos Str. 2, 08412 Vilnius, Lithuania

**Keywords:** plant microbial biostimulants, *Triticum aestivum*, *Avena sativa*, protein content, soil nitrogen, microelements, phosphorus, organic carbon, humus

## Abstract

The aim of this study was to determine the influence of microbial biostimulants on wheat and oat growth, grain yield, and grain quality and to evaluate the influence of these probiotics on some soil agrochemical traits in the open field. Active concentrations of ProbioHumus and NaturGel and their mixtures were selected under laboratory conditions using winter wheat as a reference plant. Probiotics had a biostimulating effect on the development of the underground and aboveground part of winter wheat when 2 µL/g was used for seed priming and 2 mL/100 mL for seedling spraying. Under field conditions, after treatment of soil (2 L/ha), wheat and oat seeds (2 L/t), and plants (2 L/ha) with ProbioHumus and NaturGel, it was found that the yield of the studied cereals increased, on average, by 0.50 t/ha to 1.09 t/ha. ProbioHumus promoted protein accumulation in the investigated cereal grains. The level of microelements in wheat and oat grains increased after treatment of plants with NaturGel. Probiotics improved soil agrochemical properties, such as total and nitrate nitrogen, total and available phosphorus, organic carbon, humic acid, and humus content. In conclusion, plant probiotics can be used as an ecological alternative for growing cereals and improving the agrochemical properties of the soil.

## 1. Introduction

Increasing the productivity of agricultural crops has been, and remains, one of the most important challenges for agricultural production and science. The use of chemical fertilizers to support crop production has led to the global pollution of soil, water and air. With the latest input-intensive farming systems and technologies, the use of chemical fertilizers (consisting of N, P or K) has been too high to meet the nutrient requirements of plants to increase agricultural productivity worldwide [1]. On the other hand, low fertilizer use efficiency means that only a limited amount of these nutrients are taken up by plants (30–40%) and the rest are released into the soil, thus polluting the environment. Moreover, organic farms can only fertilize the soil with certain fertilizers and to a very limited extent, so the main problem for these farms is low production yields and constantly deteriorating soil quality [2]. Today’s farming conditions are determined not only by new crop production technologies but also by soil fertility and degradation. Organic matter (humus) in the soil is rapidly declining due to active agricultural activities, as only plant residues become its source. The mass and chemical composition of these residues affect not only the humus stock but also its qualitative composition [3]. Sustainable agriculture has increased in recent times, leading to the necessity of new technological developments to reduce environmentally harmful chemical fertilizer and pesticide use [4,5]. As a result, the use of biostimulants in sustainable farming practices has become an innovative and environmentally friendly technology that improves not only yields but also soil quality [6]. Although the use of biostimulants containing live microorganisms is one of the practices used for productivity regulation, there is relatively little research on crop production and even less evidence on the potential effects of probiotics on soil properties [7,8,9]. When biostimulants are used as seed, plant or soil inoculants, they proliferate and participate in nutrient cycling and improve crop productivity [10]. Biostimulants contain different types of microorganisms that can convert important nutrients from being unavailable to available through biological processes in the soil [11,12,13]. The addition of growth-promoting micro-organisms creates an ecological balance of microflora dominated by beneficial bacteria, creating a healthier and more vibrant environment for plants and thus reducing the negative effects of excess nitrogen emissions on the soil [14,15]. It is thought that certain micro-organism referred to as “probiotics” may benefit the plant by acting as biological control agents. Higa and Parr developed the concept of effective microorganisms (EM). According to their research, the incorporation of EM into the soil, and the exposure of plants to their cultures, can improve crop growth, yield and quality [16]. This work has paved the way for the concept that plant probiotic microorganisms (PPM) can be a viable alternative to improve soil structure and quality while maintaining plant productivity [17]. PPM produced commercially by companies are being investigated in the open field for their ability to ensure yield and quality [18]. Biostimulants have shown potential in many agricultural crops, but the efficacy and specificity of probiotic products in improving cereal yields and soil structure are not well studied. There are few studies on the effects of PPM on vegetable and fruit yields, and particularly on the accumulation of bioactive compounds [19,20,21]. Knowledge of the effect of probiotics on growth, crop productivity and yield quality in wheat (*Triticum aestivum* L.) and oats (*Avena sativa* L.) is very limited. Wheat is undoubtedly a nutritionally and economically important cereal which is grown in countries all over the world. The treatment of wheat and barley seeds with Bacillus sp. has been shown to improve root growth and plant development, to increase crop yields and improve soil structure [22]. Oats (*Avena sativa* L.) are the sixth largest cereal crop in the world in terms of planted area, after wheat, maize, rice, barley and sorghum. Oats are rich in nutrients, such as fat, protein, vitamins and fiber. Exogenous application of seaweed extract has been shown to have positive effects on crop growth, yield and quality [23]. We found some evidence that the growth response of oat plants to some biostimulants and poorly soluble fertilizers was altered as a result of increased soil microbial diversity and better plant access to less soluble nutrients. The combined use of biofertilizers and rotted straw was shown to result in the highest oat productivity, especially in terms of grain yield and dry biomass production [24]. Accordingly, there is a need to investigate the influence of microbial biostimulants on crop growth and development and to apply them in modern agriculture. In this respect, the search for probiotic biostimulants that promote the growth of wheat and oats and improve crop productivity without adverse environmental impacts is a promising crop production strategy.

We hypothesized that the use of the PPM compositions ProbioHumus and NaturGel may improve soil structure and have a positive effect on cereal crop productivity. Therefore, we investigated the effect of ProbioHumus and NaturGel on cereal yield and grain quality in the open field and evaluated the effects of these biostimulants on soil agrochemical properties. The main objectives were: (1) the investigation of the impact of ProbioHumus and NaturGel on winter wheat and oat yield and quality, and (2) to evaluate the effect of probiotics on soil total and nitrate nitrogen, total and available phosphorus, organic carbon, humic acid, and humus content.

## 2. Materials and Methods

### 2.1. Plant Material and Treatments

The plant material used for the trials was winter wheat (*Triticum aestivum* L.) cv. ‘Kena DS’ and spring oats (*Avena sativa* L.) cv. ‘Symphony’. The probiotics ProbioHumus and NaturGel were used as biostimulants to improve soil properties and crop productivity and quality. The studies were carried out under controlled laboratory and field conditions on an organic commercial farm located in the Vilkaviškis district, 54°59′ N, 23°27′ E, Lithuania. Two experiments were carried out on the farm between 2017 and 2019. The plot area in the field experiment was 1 ha for each experimental variant. The first experiment analyzed the effect of probiotics on wheat growth using four different plots (4 × 1 ha) for four test variants. The season of this experiment lasted from September 2017 to August 2018. The same fields were subsequently used in a second experiment on oats, conducted from April 2019 to August 2019. All the fields used had soils belonging to the endocalcari-epihypogleyic cambisol (USDA classification), with a characteristic pH of 7.2 and a total nitrogen content of 1.54 g/kg in the arable layer at the beginning of the experiment.

#### 2.1.1. Probiotic ProbioHumus

ProbioHumus (purchased from Baltic Probiotics, Rucavas pagasts, Latvia) is a commercial probiotic containing *Bacillus subtilis*, *Bifidobacterium animalis*, *B. bifidum*, *B. longum*, *Lactobacillus diacetylactis*, *L. casei*, *L. delbrueckii*, *L. plantarum*, *Lactococcus lactis*, *Streptococcus thermophilus*, *Rhodopseudomonas palustris*, *R. sphaeroides*, and *Saccharomyces cerevisiae*.

#### 2.1.2. Probiotic NaturGel

NaturGel is a commercial probiotic preparation (purchased from Sadera, Vilnius, Lithuania) containing microorganisms of the genera *Azotobacter*, *Bacillus*, *Rhizobium*, *Bradyrhizobium*, *Lactobacillus*, and *Trichoderma*, enzymes, vitamins (B_1_, B_2_, PP, E, A), carotenoids, fulvic, humic, and amino acids, carbohydrates, as well as nitrogen, phosphorus, potassium, copper, zinc, manganese, magnesium and iron.

### 2.2. Determination of the Active Concentration of Probiotics

In order to determine the active concentration of probiotics for root development, winter wheat seeds were primed with probiotic preparations (except the control group) according to the following scheme: (1) Control (H_2_O); (2) ProbioHumus (1 µL/g); (3) NaturGel (1 µL/g); (4) ProbioHumus (1 µL/g) plus NaturGel (1 µL/g); (5) ProbioHumus (2 µL/g); (6) NaturGel (2 µL/g);( 7) ProbioHumus (2 µL/g) plus NaturGel (2 µL/g); (8) ProbioHumus (4 µL/g); (9) NaturGel (4 µL/g); and (10) ProbioHumus (4 µL/g) plus NaturGel (4 µL/g). Seeds were sown in 10 × 10 cm pots with a peat substrate. Plants were grown in a Climacell plant growth chamber (Medcenter Einrichtangen, GmbH, Planegg, Germany) at a constant temperature of 24 °C, 60 mol m^2^ s^−1^ illumination, 16/8 h photoperiod and 65% humidity. Each experimental unit consisted of 30 seeds with 10 seeds per pot. Root morphometric measurements: root length, the number of lateral roots, fresh and dry mass were taken after 16 days of growth using a ruler and balances (Kern EWJ, Germany and Sartorius BP 110S, Korea), respectively.

**Figure 1:**
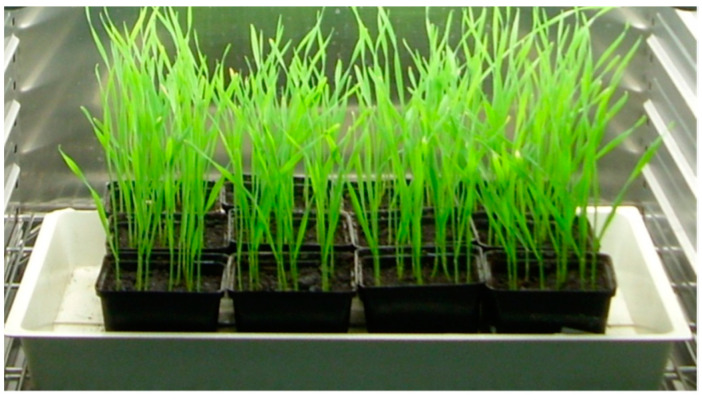
Tested wheat plants in the growing chamber after 20 days of growth.

To determine the active concentration of the probiotic for the growth and development of winter wheat seedlings, the seeds were primed with the selected concentration of the probiotic and grown as described in the previous paragraph. At BBCH-scale (code of phenologically similar growth stages of plants—the abbreviation derives from Biologische Bundesanstalt, Bundessortenamt and CHemical industry) 4–5 in the fourth to fifth leaf stage [25], the leaves of the plants were sprayed with aqueous solutions of the preparations according to the following scheme: (1) Control (H_2_O); (2) ProbioHumus (1 mL/100 mL); (3) NaturGel (1 mL/100 mL); (4) ProbioHumus (1 mL/100 mL) plus NaturGel (1 mL/100 mL); (5) ProbioHumus (2 mL/100 mL); (6) NaturGel (2 mL/100 mL); (7) ProbioHumus (2 mL/100 mL) plus NaturGel (2 mL/100 mL); (8) ProbioHumus (4 mL/100 mL); (9) NaturGel (4 mL/100 mL); (10) ProbioHumus (4 mL/100 mL) plus NaturGel (4 mL/100 mL). Wheat was harvested after 20 days of cultivation (Figure 1) and the morphometric parameters of 30 plants were measured immediately.

### 2.3. Field Experiments

The soil of the tested fields was treated with probiotics three times: (1) in the autumn of 2017 before the sowing of winter wheat, (2) in the autumn of 2018 after wheat harvest, and (3) in the spring of 2019 before the sowing of oats. The preparations of 2 L/ha were diluted 1:100 with water and the soil of fields (4 × 1 hectare) were sprayed according to the scheme: (1) Control group without any treatment, (2) ProbioHumus, (3) NaturGel, and (4) ProbioHumus and NaturGel in combination. Before sowing, winter wheat seeds, except for the control group, were primed with probiotic preparations ProbioHumus (2 L/t), NaturGel (2 L/t) and ProbioHumus with NaturGel (2 L/t + 2 L/t) in combination. The probiotic treatment of oat seeds was performed in the spring of 2019 using the same doses. Wheat and oat plants were sprayed twice during the growing season with these products (according to the same schedule) at the nine-leaf stage (BBCH 18–19) and at the beginning of stem elongation (BBCH 32–37) [25]. The probiotics were diluted 1:200 with water at 2 L/ha and sprayed on plants. At the end of the flowering stage (BBCH 58–59), crop height was measured. Before harvesting at full maturity, a random sample of 30 plants was taken from each experimental variant for measurements of yield productivity indices including the number of grains per plant, the average grain weight per plant and the 1000 grain weight. After harvesting with a combine harvester, the yield of wheat and oats was estimated in t/ha.

### 2.4. Determination of Photosynthetic Pigments

Wheat and oat leaf samples were taken for analysis at the beginning of stem elongation (BBCH 56–57 [25]). Three leaves per plant of thirty wheat and oat plants were used to determine photosynthetic pigments. Leaf fragments (up to 50 mg) were extracted with *N*,*N*-dimethylformamide at 4 °C for four days. The extract was filtered, and the absorbance was measured by spectrophotometer at 480 nm, 647 nm and 664 nm. The chlorophyll a (C_a_), chlorophyll b (C_b_) and total carotenoid (C_c_) content was calculated according to [26], using the formulas:

C_a_ = 11.65 × A_664_ − 2.69 × A_647_, (1)

C_b_ = 20.81 × A_647_ − 4.53 × A_664_, (2)

C_c_ = (1000 × A_480_ − 0.89 × C_a_ − 52.02 × C_b_)/245, (3)

where A—absorption, C—concentration in mg/L.

The quantity of pigments of fresh weight per unit was calculated by the following formula:

P = ((C × V) × dilutions times)/M × 1000,(4)

where P—pigment content in mg/g of fresh mass, V—pigment extract volume, M—fresh mass in grams.

### 2.5. Estimation of Cereal Protein Content

Grain samples from 30 wheat and oat plants were milled to avoid sample inhomogeneity and 0.3 g of each test variant was used for the measurements with three replicates. The protein content was determined by an improved Kjeldahl method which can be divided into three steps: digestion, neutralization and titration. A Kjeldahl protein-nitrogen analyzer, consisting of a graphite digestion complex SH220N with exhaust system S402 and automatic distiller K9840 (Hanon Instruments, Jinan, China), and an automatic potentiometric titrator KEM AT-7100 (Kyoto Electronics, Kyoto, Japan) were used. Cereal protein was calculated by multiplying the nitrogen content by 5.7.

### 2.6. Determination of Mineral Content of Cereals

Samples of grains were prepared as described in Section 2.5. The content of Mn, Fe, and Zn in oat and wheat grains was determined by standard atomic absorption spectrometry (AAS) using a spectrometer (Z-8200, Hitachi, Tokyo, Japan), following mineralization in a mixture of concentrated hydrochloric and nitric acids in a 1:3 ratio.

### 2.7. Soil Sampling

Soil samples for chemical analyses and tests were taken twice: before the installation of the test fields (8 August 2017) and at the end of the study (8 August 2019). For the experiments, a 1 × 4 ha study area was set up and 80 soil sampling points were marked by GPS (20 samples from the control area and 20 samples from each probiotic plot). Soil 20 cm deep and 2.5 cm in diameter was collected with probe punctures and mixed to form a single composite soil sample and then stored in a plastic box at 4 °C until further analysis.

### 2.8. Chemical Analysis of Soil

The soil samples were crushed, sieved through a 2 mm sieve, homogeneously mixed and dried in an oven at 105 °C for 16 h and the following soil agrochemical parameters were determined:

#### 2.8.1. Total Nitrogen

Total nitrogen (N) concentrations were estimated after mineralization with sulfuric acid in the presence of potassium sulphate and low copper concentrations (Kjeltabs Cu/3.5, Kjeltabs KPC/4.5, Catalyst, Changsha, China) by the Kjeldahl method [27], using the same analyzer system as described in Section 2.5.

#### 2.8.2. Nitrate and Ammonia Nitrogen

Nitrate and ammoniacal nitrogen levels in the soil sample were measured photometrically according to the national standard ISO 14256-2:2005. The soil sample was extracted with 1:2.5 1 M KCl. The nitrate and ammoniacal nitrogen content of the filtrate obtained was determined using a Fiastar 5000 Analyser (FOSS, Hillerd, Danmark).

#### 2.8.3. Total Phosphorus

Total phosphorus content was obtained by digestion of 0.25 g of soil previously treated in 5 mL of hydrofluoric acid (40%) and 1.5 mL of HClO_4_ (65%) [28] followed by molybdate spectrophotometric measurements.

#### 2.8.4. Available Phosphorus

Available phosphorus (P_2_O_5_) was extracted with 0.5 M NaHCO_3_ and analyzed spectrophotometrically at 880 nm according to the method described by Olsen [29].

#### 2.8.5. Organic Carbon

Soil organic carbon content was determined by the dichromate oxidation method spectrophotometrically at 590 nm (Analytik Jena Specord 210 plus, Jena, Germany) using glucose as a standard after wet combustion [30].

#### 2.8.6. Humus

The obtained data on C_org_ were converted into humus content in the soil using the following formula: humus content = 1724 × C_org_.

#### 2.8.7. Humic Acids

Soil samples were weighed and sodium pyrophosphate alkali solvent was added and put on a boiling water bath. Standard solutions containing 0.002–0.012% of humic acid were prepared in pyrophosphate solvent too. The absorbance was recorded in two wavelengths, 465 and 665 nm [31], and used to calculate humic acid values.

### 2.9. Statistical Analysis

Analysis of variance (one-way ANOVA) was performed. Tukey’s HSD post hoc test was used to compare means. Differences with *p*-values < 0.05 were considered significant. Different lower case letters indicate statistically significant differences (*p* < 0.05). Error bars indicate the standard deviation of the mean.

## 3. Results

### 3.1. Impact of Probiotics on Wheat Root Formation under Controlled Conditions

Root morphometric measurements showed that root development was most intense when the seeds were treated with 2 µL/g probiotics. The highest average fresh root weight was obtained in the experimental variant with ProbioHumus and ProbioHumus + NaturGel, with increases in root weight of 28% and 21%, respectively, compared to the control plants. Both probiotic preparations promoted dry matter accumulation (Table 1). The root length was 22% longer in the experimental variant with ProbioHumus (2 µL/g). The number of lateral roots increased by 17% and 10% after treatment with ProbioHumus (2 µL/g) and ProbioHumus (1 µL/g) + Naturgel (1 µL/g), respectively, compared to the control group (Table 1).

### 3.2. Impact of Probiotics on Wheat Seedling Formation under Controlled Conditions after 20 Days of Cultivation

The results in Table 2 show that ProbioHumus and NaturGel were the most suitable for the aboveground part of the seedling establishment after a foliar spray of 2 mL/100 mL. This treatment resulted in a 3–2% increase in seedling length compared to the control group and an average increase in fresh and dry weight of 12–16%. In addition, we found that the complex treatment with both probiotic compositions also improved the morphometric parameters of wheat seedlings compared to the control group (Table 2).

### 3.3. Influence of Probiotics on the Growth of Wheat and Oats under Natural Field Conditions

**Figure 2:**
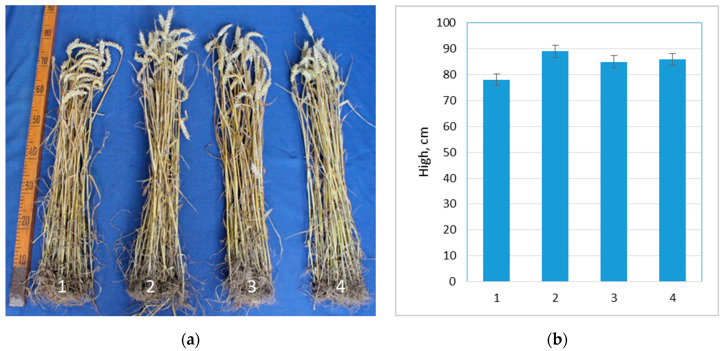
Effect of ProbioHumus and NaturGel on wheat growth: (**a**) photo of wheat before the height measurements at maturity stage; (**b**) height of wheat grown in natural field conditions. 1—Control; 2—ProbioHumus; 3—NaturGel; 4—ProbioHumus + NaturGel.

Wheat height measurements at maturity stage showed that ProbioHumus-treated plants were on average 14% taller than the control plants. NaturGel alone and in combination with ProbioHumus was less effective: wheat height increased by 9% and 10%, respectively, vs. control group (Figure 2).

**Figure 3:**
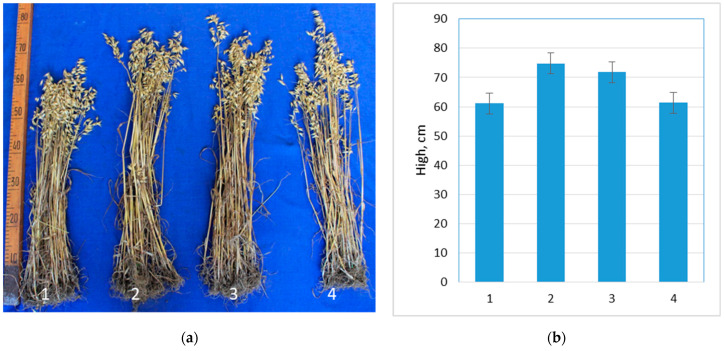
Effect of ProbioHumus and NaturGel on oats growth: (**a**) photo of oats before the height measurements at maturity stage; (**b**) height of oats grown in natural field conditions. 1—Control; 2—ProbioHumus; 3—NaturGel; 4—ProbioHumus + NaturGel.

ProbioHumus had the greatest effect on oat growth, with a 22% increase in plant height compared to the control plants (Figure 3).

### 3.4. Effect of ProbioHumus and NaturGel on the Accumulation of Photosynthetic Pigments in Wheat and Oat Leaves

Quantitative analysis of wheat leaf pigments showed that the highest chlorophyll content (2.77 mg/g FM) was found in wheats treated with ProbioHumus. The tested treatments did not significantly affect the carotenoid accumulation in wheat leaves vs. control group. In contrast, the evaluation of pigment accumulation in oat leaves showed that all the treatments promoted the accumulation of pigments in oats’ leaf tissues. The highest levels were found in oats treated with ProbioHumus in combination with NaturGel, with an increase of 11% in chlorophyll and 20% in carotenoids compared to the control plants (Table 3).

### 3.5. Effect of ProbioHumus and NaturGel on the Formation and Yield of Productivity Elements in Wheat and Oats

In the experimental treatments with ProbioHumus and ProbioHumus + NaturGel, the grain weight per thousand grains increased by 11% and 15%, respectively, compared to the control group. A significant yield increase of 0.8 t/ha was obtained with NaturGel at 11.1 t/ha. In contrast, the control field yielded 10.3 t/ha. The treatment of wheat with ProbioHumus in combination with NaturGel also increased yield by 0.8 t/ha (Table 4).

Studies on the effect of probiotics on the formation of productivity elements in oats showed that ProbioHumus had the highest activity, with a 17% increase in the number of oat kernels in the panicle, a 14% increase in the weight of one thousand kernels, and an 11% increase in the weight of grains per plant, compared to control plants. Yield increases (t/ha) were found in all treatments: 1.0 t/ha with ProbioHumus, 0.50 t/ha with NaturGel and 0.80 t/ha with NaturGel + ProbioHumus (Table 5).

### 3.6. Effect of Probiotics on Protein and Micronutrient Content of Wheat and Oat Grains

**Figure 4:**
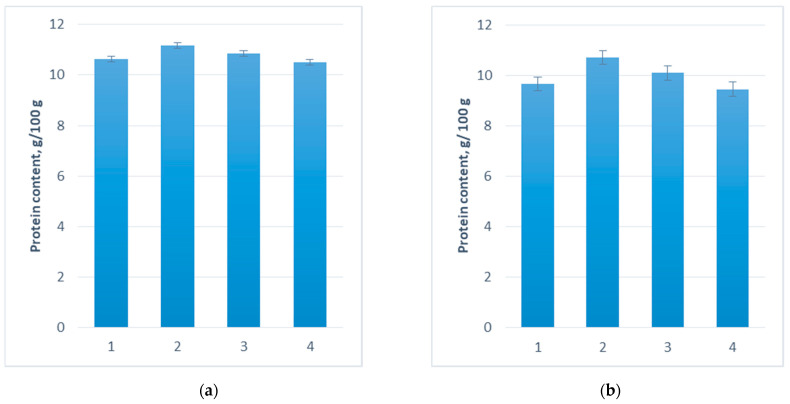
Effect of NaturGel and ProbioHumus on the protein content of wheat (**a**) and oat (**b**) grains. 1—Control; 2—ProbioHumus; 3—NaturGel; 4—ProbioHumus + NaturGel.

The analysis of the protein content of wheat grain showed that ProbioHumus-treated fields were more protein rich. The protein content increased by 5% (Figure 4a). In comparison, oat grains harvested from ProbioHumus treated plants showed an increase in protein content of 1.04 g/100 g. NaturGel treatment increased protein accumulation in oat grain by 5% (Figure 4b).

The study of microelement content in wheat grain showed that the NaturGel treated plant showed an increase in Mn and Zn concentrations of 0.5 and 1.6 mg/kg, respectively. In the ProbioHumus variant, the concentration of iron was higher by 1.5 mg/kg. In contrast, the Fe concentration in oat grain was not changed by the application of probiotics, but the Mn and Zn content of oat grain increased by 0.4 and 0.7 mg/kg, respectively, after NaturGel application. A similar effect was observed when NaturGel was used in combination with ProbioHumus (Table 6).

### 3.7. Influence of Probiotics on Soil Agrochemical Indicators

At the beginning of the experiment, before the effect of the probiotics on the agrochemical properties of the soil was investigated, the nitrate-nitrogen content of the soil was 6.4 mg/kg (Table 7). In the control plots, nitrate-nitrogen content remained almost the same during the two years of the experiment. After two years of probiotic treatment, the level of this indicator increased by 10–25% compared to the 2017 and 2019 controls. In contrast, in 2019, the ammoniacal nitrogen content of the soil decreased after two years of soil and plant treatment with probiotics: it decreased by 0.49, 0.39, and 0.13 mg/kg in the experimental plot with ProbioHumus, NaturGel and ProbioHumus combined with NaturGel, respectively, compared to the 2017 controls. Measurements of total nitrogen (N) (0–40 cm in the topsoil layer) showed that the probiotic treatment did not have a significant effect on this agrochemical parameter. ProbioHumus + NaturGel significantly increased the total phosphorus content of soil samples by 8% vs. control. The use of probiotics affected total phosphorus mineralization and affected phosphorus availability to plants. The most effective treatment, with a 20-fold increase, was ProbioHumus in combination with Naturgel. The total organic carbon content of plots treated with ProbioHumus increased by 9%. This was slightly higher in the other probiotic treated plots compared to the control. The humic acid content was not affected by the probiotics. The average difference in humus content between the control and ProbioHumus treated field soil was 10%, while with Probiotic + NaturGel and NaturGel it was 7% and 4%, respectively.

## 4. Discussion

Organic farming, which strictly prohibits the use of synthetic fertilizers, produces products with improved nutritional qualities, but low yields and persistent degradation of soil quality [2]. Biostimulants have been identified as an alternative to increase soil fertility and crop production in sustainable agriculture. The use of microbial and non-microbial plant biostimulants is a promising strategy in this respect, as they stimulate plant growth and improve crop performance, making them environmentally friendly, cost-effective, non-toxic and adaptable; they also help to maintain soil structure and biodiversity of agricultural land [3,32,33]. PPM and non-microbial plant biostimulants are commonly used for crops grown in the open field and in greenhouses, including fruit trees, berries, grapes, vegetables, and ornamentals [34,35]. Most of the work in this area has been carried out with complex mixtures of products, such as plant or seaweed extracts and recycled waste [36,37]. In the current study, the PPM compositions ProbioHumus, NaturGel and their mixture were tested. Probiotic mixtures were used in our experiment because the combination of several beneficial preparations can be more effective than the use of a pure active ingredient, especially if they act synergistically. The scientific literature has confirmed that the combined use of non-microbial biostimulants resulted in the highest crop productivity, especially in terms of grain yield and dry biomass production [38,39], and it has also been reported that the combination of composted straw and biofertilizers significantly increased rice yield [40].

Under laboratory conditions, we selected a concentration of probiotics and their mixtures with a biostimulatory effect on the growth and development of winter wheat at a concentration of 2 mL/100 mL. The use of probiotics at the selected concentrations confirmed the accelerated growth of wheat and oats under natural field conditions. ProbioHumus had the most marked effect on the growth of oats, with a 22% increase in plant height compared to the control plants, and an average increase of 14% in wheat treated with ProbioHumus. According to scientific data, increased plant growth with non-microbial inoculants was in some cases associated with increased chlorophyll and carotenoid content and improved plant nutrient status [41]. It has been reported in the literature that Kelpak treatment of seaweed resulted in a 10% increase in chlorophyll content compared to control plants [42]. Quantitative analysis of chlorophyll and carotenoids in leaves revealed the influence of the compounds studied on changes in the pigment composition of wheat and oats. The highest chlorophyll values (2.77 mg/g FM) were found using ProbioHumus. The investigated preparations did not show any significant effect on carotenoid accumulation in wheat leaves.

When investigating the formation of cereal productivity, it was found that the highest effect on wheat yield was obtained in the experimental variants with ProbioHumus and ProbioHumus + NaturGel, resulting in increases in thousand grain weight of 11 and 15%, respectively. ProbioHumus combined with NaturGel increased wheat yield by 1.09 t/ha. The increase in oat yield was found in all treatments: ProbioHumus 1.0 t/ha, NaturGel 0.50 t/ha and NaturGel + ProbioHumus 0.80 t/ha. Improving seed yield and grain protein concentration are two major challenges in cereal production, as these traits are dominant determinants of the economic value of the harvested product [43]. The protein content of wheat and oat grains was analyzed and it was found that ProbioHumus-treated wheat grains matured with a higher protein content, i.e., an increase of 5% in protein content compared to the control plants. In contrast, oat grains harvested from ProbioHumus-treated plants showed an increase in protein content of 1.04 g/100 g. In addition, the quality of the cereals is determined by their mineral composition. Cereals have been shown to be an essential source of Fe and Zn in the daily diet of humans [44,45], but the concentrations of these minerals in flour are generally low [46]. We found that the trace elements Mn and Zn were increased in wheat and oat grains grown in fields treated with NaturGel: 0.5 and 1.6 mg/kg in wheat and 0.4 and 0.7 mg/kg in oats. The increase in Fe concentration (1.5 mg/kg) was observed only in the wheat grain samples taken from the ProbioHumus treatment. The effect of other probiotics on the accumulation of the tested trace elements was not statistically significant but had a positive effect on the increase in these elements in wheat and oat grains.

Literature reports show that the use of bioactive natural materials and microbial inoculants can not only improve crop production, but also represents a valuable tool for improving soil quality [47,48]. Soil agrochemical properties that are important for plant growth include humus, total nitrogen and plant available phosphorus, and humic acids and organic matter [49]. These components are not only important sources of material for the development of plant tissue structures but are also actively involved in plant metabolism. Nitrogen is required for all plant growth processes [50]. In terms of total nitrogen, arable soils (0–40 cm in the topsoil) are divided into groups of low nitrogen up to 0.2%, medium nitrogen up to 0.21–0.3%, sufficient nitrogen up to 0.31–0.4% and high nitrogen of more than 0.4% [51]. In our field trial, the agrochemical properties of the ploughsoil after treatment with probiotics changed according to the total nitrogen from the low to medium nitrogen group. In contrast, in the ProbioHumus treated field, the ammoniacal nitrogen content of the soil decreased by 0.49 mg/kg. Nevertheless, there was a tendency for the nitrate and nitrogen content to increase in the soil treated with ProbioHumus and ProbioHumus in combination with NaturGel.

Probiotics containing live microorganisms are one of the management practices that can help maintain or increase phosphorus levels and improve soil quality in arable soils. Phosphorus is an essential component of many vital plant processes. Insufficient phosphorus in the soil leads to poor plant development and growth, as well as leaf curling [52]. Total phosphorus in soil can range from 112 to 600 mg/kg [53]. In our experimental fields, it ranged from 269 to 310 mg/kg. Phosphorus that is immobile and unavailable to the plant for uptake accounts for 80%. Microorganisms are known to play a key role in the processing and conversion of organic forms of phosphorus into plant-available forms [54]. We found that all the probiotics we tested had a significant effect on the available phosphorus in the soil, with ProbioHumus together with NaturGel having the largest effect. The fact that NaturGel contains phosphorus in an available form may also be important, but the beneficial effect of ProbioHumus containing live micro-organisms on phosphorus mineralization was demonstrated by an 18-fold increase in the amount of available phosphorus in the ProbioHumus treated plots vs. control.

Humic acids, which are the main component of soil humus, indicate the properties of humus in the natural environment [55]. It is known that the quantity of humic acids present in humus and their properties change with the application of biostimulants [56]. We have shown that the humic acid content was slightly affected by probiotic use.

Organic carbon is one of the main indicators of soil quality. Even a small increase in organic carbon can activate the soil and affect the potential soil fertility. There is no consensus on the influence of the technologies used on the organic carbon content of the soil [57]. This effect depends on soil type, inputs, etc. In our study, the total organic carbon content of plots treated with the probiotic ProbioHumus increased by 9% compared to the control plot. The other probiotic-treated plots showed a slightly lower rate. Microbial activity is important for several soil reactions and functions, including organic matter decomposition and humus formation [56,58]. In total, 80–85% of soil organic matter is composed of humus. Humus not only improves soil structure and the water and weather regime but is also an important source of microorganisms and plant nutrients. Conflicting results have been obtained concerning the influence of biostimulants on organic matter content [59]. In Lithuania, the humus content varies from 1.5 to 2.76% in different regions of the country [60]. This soil quality indicator was quite high in our field trials, reaching 2.32% in untreated plots and 2.63% in ProbioHumus-treated plots.

In conclusion, the data presented in this study clearly show that the microbial biostimulants ProbioHumus, NaturGel, and their mixture, had a positive effect on the growth of wheat and oats and on the formation of productivity elements. ProbioHumus in combination with NaturGel increased wheat yield by 1.09 t/ha. A yield increase in oats was found for all treatments: ProbioHumus 1.0 t/ha, NaturGel 0.50 t/ha and NaturGel + ProbioHumus 0.80 t/ha. The application of probiotics affected the quality of the cereal yield: ProbioHumus promoted protein accumulation by 5% and 8% in wheat and oat grains, respectively. The content of the microelements Mn and Zn in wheat and oat grains changed after NaturGel treatment. ProbioHumus had the most marked effect on the Fe concentration (1.5 mg/kg increase) in wheat grains. Soil chemical analysis showed that probiotics improved agrochemical properties, such as total and nitrate nitrogen, total and available phosphorus, organic matter, humic acid and humus content. Therefore, microbial probiotics should be further investigated and used in the development of sustainable agriculture as an ecological alternative for crop growth, grain yield, quality and potential soil fertility.

## Figures and Tables

**Table 1:** Effect of seed priming with probiotics on wheat root morphometric parameters after 16 days of cultivation under controlled laboratory conditions. Photo shows the effect of the selected concentration of probiotics (2 µL of probiotic on 1 g of seeds) on the development of wheat roots: 1. Control; 2. ProbioHumus; 3. NaturGel; 4. ProbioHumus + NaturGel.

Treatments	Primary Root Length (cm)	Average Mass (g)		Number of Lateral Roots	
		Fresh	Dry		
Control (H_2_O)	30.0 ± 0.35 b	0.14 ± 0.011 b	0.0133 ± 0.058 b	30 ± 2.2 b	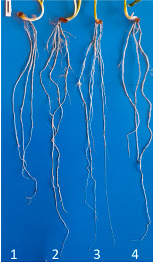
Treatment (1 µL/g)					
ProbioHumus	32.5 ± 1.06 b	0.15 ± 0.007 b	0.0134 ± 0.003 b	31 ± 1.5 b	
NaturGel	31.0 ± 0.71 b	0.13 ± 0.005 b	0.0132 ± 0.002 b	32 ± 1.8 b	
ProbioHumus + NaturGel	27.0 ± 1.40 b	0.14 ± 0.004 b	0.0134 ± 0.001 b	31 ±1.6 b	
Treatment (2 µL/g)					
ProbioHumus	36.5 ± 1.09 a	0.18 ± 0.009 a	0.0139 ± 0.001 a	35 ± 1.5 a	
NaturGel	33.0 ± 0.82 b	0.16 ± 0.004 a	0.0137 ± 0.003 a	33 ± 2.0 ab	
ProbioHumus + NaturGel	32.0 ± 1.31 b	0.17 ± 0.003 a	0.0136 ± 0.001 a	33 ± 1.6 ab	
Treatment (4 µL/g)					
ProbioHumus	29.5 ± 1.11 b	0.13 ± 0.003 b	0.0132 ± 0.003 b	31 ± 1.5 b	
NaturGel	28.0 ± 0.65 b	0.10 ± 0.003 c	0.0122 ± 0.004 c	29 ± 1.8 b	
ProbioHumus + NaturGel	27.0 ± 1.52 b	0.12 ± 0.004 b	0.0124 ± 0.001 c	30 ± 1.4 b	

Values reported are mean of thirty roots with standard deviation. Means with different letters in the same column are significantly different (*p* < 0.05).

**Table 2:** Effect of leaf-sprayed probiotic preparations on morphometric parameters of winter wheat seedlings (per plant) grown under controlled laboratory conditions.

Treatment	Average Length (cm)	Average Mass (g)	
		Fresh	Dry
Control (H_2_O)	38.19 ± 0.31 b	0.573 ± 0.05 b	0.083 ± 0.01 b
Treatment (1 mL/100 mL)			
ProbioHumus	38.28 ± 0.23 b	0.582 ± 0.06 b	0.084 ± 0.01 b
NaturGel	38.26 ± 0.13 b	0.577 ± 0.03 b	0.087 ± 0.01 b
ProbioHumus + NaturGel	38.24 ± 0.31 b	0.579 ± 0.02 b	0.089 ± 0.01 ab
Treatment (2 mL/100 mL)			
ProbioHumus	39.41 ± 0.32 a	0.629 ± 0.02 a	0.098 ± 0.03 a
NaturGel	39.32 ± 0.21 a	0.622 ± 0.02 a	0.095 ± 0.03 a
ProbioHumus + NaturGel	39.38 ± 0.21 a	0.631 ± 0.02 a	0.099 ± 0.03 a
Treatment (4 mL/100 mL)			
ProbioHumus	38.29 ± 0.23 b	0.589 ± 0.02 b	0.089 ± 0.02 b
NaturGel	38.26 ± 0.13 b	0.582 ± 0.03 b	0.091 ± 0.01 b
ProbioHumus + NaturGel	38.38 ± 0.13 b	0.589 ± 0.01 b	0.093 ± 0.01 b

Values reported are mean of thirty seedlings with standard deviation. Means with different letters in the same column are significantly different (*p* < 0.05).

**Table 3:** Effect of ProbioHumus and NaturGel on pigment accumulation in wheat and oat leaves at the beginning of stem elongation.

Test Variant	Chlorophyll a	Chlorophyll b	Chlorophyll a + b	Carotenoids
Wheat	mg/g FW			
Control	1.32 ± 0.11 a	0.71 ± 0.05 a	2.03 ± 0.17 a	0.22 ± 0.02 a
ProbioHumus	1.44 ± 0.09 b	1.32 ± 0.09 b	2.77 ± 0.18 b	0.21 ± 0.01 a
NaturGel	1.31 ± 0.11 a	0.92 ± 0.09 ab	2.22 ± 0.16 a	0.13 ± 0.01 b
NaturGel + ProbioHumus	1.31 ± 0.10 a	1.23 ± 0.12 b	2.13 ± 0.19 a	0.15 ± 0.02 b
Oats				
Control	1.17 ± 0.11 a	0.42 ± 0.03 a	1.60 ± 0.14 a	0.25 ± 0.01 a
ProbioHumus	1.19 ± 0.12 a	0.44 ± 0.03 a	1.63 ± 0.12 a	0.27 ± 0.01 a
NaturGel	1.24 ± 0.10 a	0.46 ± 0.04 a	1.66 ± 0.12 a	0.23 ± 0.01 a
NaturGel + ProbioHumus	1.30 ± 0.25 a	0.49 ± 0.04 a	1.79 ± 0.14 b	0.30 ± 0.01 b

Values reported are mean of three experimental repeats with standard deviation. Means with different letters in the same column and the same cereals are significantly different (*p* < 0.05).

**Table 4:** Effect of probiotic preparations on wheat grain yield formation at full maturity stage.

Test Variant	Number of Grain per Ear	Grain Weight (g per Plant)	Thousand Grain Weight (g)	Yield (t/ha)
Control	30 ± 2.2 b	3.4 ± 1.1 b	40 ± 0.31 b	10.3 b
ProbioHumus	32 ± 2.0 b	3.6 ± 1.2 b	44 ± 0.36 a	10.8 a
NaturGel	34 ± 1.4 b	3.8 ± 1.0 b	45 ± 0.72 a	11.1 a
ProbioHumus + NaturGel	34 ± 1.3 b	3.9 ± 0.5 b	46 ± 0.41 a	11.1 a

Values reported are mean of thirty crops with standard deviation. Means with different letters in the same column are significantly different (*p* < 0.05).

**Table 5:** Effect of probiotic preparations on oat panicle growth and grain yield at maturity stage. The photo shows the effect of probiotics on the development of oat panicles: 1. Control; 2. NaturGel; 3. ProbioHumus; 4. ProbioHumus + NaturGel.

Test Variant	Number of Grains per Panicle	Grain Weight (g per Plant)	Thousand Grain Weight (g)	Yield (t/ha)	
Control	63 ± 2.2 b	2.8 ± 0.2 a	34 ± 0.33 b	7.5 b	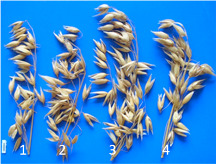
ProbioHumus	74 ± 1.4 a	3.2 ± 0.4 a	38 ± 0.72 a	8.5 a	
NaturGel	64 ± 2.5 b	2.8 ± 0.1 a	36 ± 0.30 a	8.0 a	
ProbioHumus + NaturGel	69 ± 1.8 ab	3.1 ± 0.1 a	37 ± 0.61 a	8.3 a	

Values reported are mean of thirty crops with standard deviation. Means with different letters in the same column are significantly different (*p* < 0.05).

**Table 6:** Effect of probiotics on micronutrient content of wheat and oat grains.

Test Variant	Mineral Content, mg/kg		
Wheat	Iron	Manganese	Zinc
Control	18.2 ± 0.6 a	3.3 ± 0.0 a	17.1 ± 0.6 a
ProbioHumus	19.7 ± 0.1 b	3.2 ± 0.0 a	17.7 ± 0.8 a
NaturGel	18.5 ± 0.2 a	3.8 ± 0.1 b	18.7 ± 0.6 b
NaturGel + ProbioHumus	20.3 ± 0.8 b	3.5 ± 0.1 ab	18.4 ± 0.4 b
Oats			
Control	23.2 ± 1.2 a	8.1 ± 0.0 a	10.6 ± 0.2 a
ProbioHumus	23.6 ± 1.1 a	8.2 ± 0.6 a	10.7 ± 0.0 a
NaturGel	23.3 ± 0.6 a	8.5 ± 0.3 a	11.3 ± 0.1 b
NaturGel + ProbioHumus	23.8 ± 1.2 a	8.6 ± 0.2 a	11.0 ± 0.0 b

Values reported are mean of three experimental repeats with standard deviation. Means with different letters in the same column and the same cereals are significantly different (*p* < 0.05).

**Table 7:** Effect of probiotic preparations on soil agrochemical parameters.

Agrochemical Indicators	Parameters	August 2017	August 2019			
		2017 Control	2019 Control	ProbioHumus	NaturGel	ProbioHumus + NaturGel
Nitrate-nitrogen	mg/kg	6.42 ± 0.2 a	6.1 ± 0.4 a	7.07 ± 0.56 b	7.98 ± 0.66 b	7.57 ± 0.64 b
Ammoniacal-nitrogen	mg/kg	2.27 ± 0.1 a	2.14 ± 0.14 a	1.78 ± 0.11 b	2.14 ± 0.17 a	1.87 ± 0.13 ab
Total nitrogen	%	0.154 ± 0.01 a	0.138 ± 0.02 a	0.118 ± 0.01 b	0.136 ± 0.01 a	0.123 ± 0.02 ab
Total phosphorus	mg/kg	269 ± 11 a	278 ± 10 a	291 ± 9.5 a	281 ± 14 a	310 ± 14 b
Available phosphorus	mg/kg	0.96 ± 0.08 a	1.02 ± 0.07 a	18.6 ± 1.1 b	19.3 ± 1.7 b	20.1 ± 1.9 b
Humic acids	%	0.23 ± 0.01 a	0.22 ± 0.01 a	0.22 ± 0.01 a	0.25 ± 0.01 a	0.24 ± 0.01 a
Organic carbon	%	1.34 ± 0.03 a	1.36 ± 0.10 a	2.03 ± 0.16 b	2.01 ± 0.17 b	2.02 ± 0.02 b
Humus	%	2.31 ± 0.12 a	2.32 ± 0.18 a	2.53 ± 0.17 b	2.41 ± 0.13 ab	2.48 ± 0.15 ab

Values reported are mean of three experimental repeats with standard deviation. Means with different letters in the same line are significantly different (*p* < 0.05).

## Data Availability

The data supporting the reported results can be found in the archive of scientific reports of the Nature Research Centre.
